# Clinical Impact of the ABO Blood Type in Patients with Rheumatic Diseases: Is there a Link to the ABO and Rhesus?

**DOI:** 10.31138/mjr.32.3.237

**Published:** 2021-09-30

**Authors:** Gehan I. Salem, Nada M. Gamal, Esraa A. Talaat, Dina H. El-Hammady, Nevin Hammam, Tamer A. Gheita

**Affiliations:** 1 Department of Rheumatology and Rehabilitation, Faculty of Medicine, Assiut University, Assiut, Egypt; 2 Department of Rheumatology and Rehabilitation, Faculty of Medicine, Helwan University, Cairo, Egypt; 3 Rheumatology Department, Faculty of Medicine, Cairo University, Cairo, Egypt

**Keywords:** rheumatic diseases, ABO blood groups, Rh factor

## Abstract

**Objectives:**

Several studies have shown associations of ABO and Rh blood groups with various diseases; however, the relationship of ABO and Rh blood groups with rheumatic diseases are scarce. The aim of the present study was to examine whether there is an association between ABO and Rh blood groups and the types of rheumatic diseases.

**Method:**

In this multi-centre cross-sectional study, sociodemographic data, type of rheumatic disease, and type ABO and Rh blood groups were examined for patients with different rheumatic diseases.

**Results:**

A total of 304 patients; 207 (68.1%) were diagnosed with rheumatoid arthritis, and 40 (13.2%) had systemic lupus erythematosus. The patients were assessed for blood types; 37.8% patients had A type, 27.6% had B type, 19.1% had O type, and 15.4% had AB type. The Rh (+) blood group was more prevalent (89.1%) than Rh (–). Blood group A was more prevalent in patients with rheumatic disease, followed by B, O, and AB respectively, although there was no significant difference in the distribution of ABO groups among rheumatic diseases. Female gender, smoking, and anti-cyclic citrullinated peptide are significantly different between the blood groups within rheumatic diseases.

**Conclusion:**

The A and Rh (+) blood groups were more commonly observed in patients with rheumatic diseases. There was lack of association between types of rheumatic diseases and ABO blood groups. The study provides knowledge for the interaction between ABO blood groups and several risk factors related to rheumatic diseases and may serve a guide for future clinical studies.

## INTRODUCTION

Up to 34 blood group systems have been reported with the ABO, Rhesus (Rh) being the most popular.^1^ The distribution of the four ABO blood types, A, B, AB, and O, depend on the presence or absence of the A and B antigens, also known as blood group antigens, in the red blood cells (RBCs) of populations throughout the world. Blood type O is the most common worldwide, followed by A and B, and AB being the least common.^2^ The blood groups in the Rhesus system are classified as Rh and Rh+, depending on the presence of the D antigen located on the red blood cells (RBCs) surface.^2^

The association of ABO and Rh blood groups with various diseases, such as cancer, cardiovascular disorders, infections, and diabetes mellitus, has been demonstrated.^3^ The field of red cell biology is undergoing a quiet revolution and RBCs are emerging as important modulators of the innate immune response. RBCs, with ABO antigen inside, may promote immune activation or maintain immune quiescence.^4^ RBCs are in contact with complement proteins in the blood plasma. The complement system represents the first line of defence involved in the clearance of pathogens, dying cells and immune complexes. Various complement regulatory proteins are found in plasma and on the cell membrane, and prevent complement activation on the RBCs. Decreased expression and/or function of complement regulatory proteins may result in unwanted complement activation and accelerated removal of RBCs.^5^ The immunologic function of RBCs provides an understudied and potentially rich area of research that may yield novel insights into mechanisms of immune regulation.

The revisited notion of the importance of RBCs in rheumatic diseases is largely based on their clinical and experimental associations with thrombosis or bleeding, implying a prospective therapeutic target in many disorders. In a Turkish study, the higher incidence of diverse rheumatic diseases in different blood types was associated with special genetic predisposition.^2^ Lack of association between ABO group and rheumatic diseases in China has been observed.^6^ The literature on the frequencies of the blood groups according to the rheumatic diseases is limited and scarce. Therefore, we sought to determine the association between the ABO and Rh blood groups and the type of rheumatic disease in a cohort of Egyptian patients.

## PATIENTS AND METHODS

The study included 304 patients with various rheumatic diseases who were diagnosed according to their corresponding classification criteria for rheumatoid arthritis (RA),^7^ systemic lupus erythematosus (SLE),^8^ systemic sclerosis (SSc),^9^ spondyloarthritis (SPA),^10^ idiopathic inflammatory myositis (IIM),^11^ vasculitis – a case with polyarteritis nodosa (PAN),^12^ and another with giant cell arteritis (GCA),^13^ Behçet’s disease (BD),^14^ Sjögren’s syndrome (SS),^15^ undifferentiated connective tissue disease (UCTD), and juvenile idiopathic arthritis (JIA).^16^ Informed consent was obtained from all subjects and the ethical approval was obtained from the Ethics Committee of Assiut Faculty of Medicine.

The patients were subjected to full history taking**,** and clinical examination. The family history, consanguinity, smoking (active and passive), and comorbidities such cardiovascular diseases, as diabetes mellitus, hypertension, infection, hypothyroidism, bronchial asthma, and interstitial pulmonary fibrosis (IPF) were considered. Routine laboratory markers were measured using the standard methods. The ABO and Rhesus blood systems were determined in the hospital using standard hemagglutination tests. Patients were classified according to blood groups (A, B, AB, O) and Rh status (+/–).

### Statistical analysis

Data was analysed using SPSS (Statistical Package for the Social Sciences) version 20. Results were presented as mean±SD (range) and as frequency (%). For non-parametrically distributed quantitative data, comparison between two groups was done using Mann-Whitney test and for qualitative variables Chi-square test or Fisher’s exact test were considered. Comparisons between all blood groups were conducted using ANOVA. A multivariable regression analysis of the significantly related factors was considered. A *p*-value <0.05 was considered significant.

## RESULTS

### Patients’ characteristics

The study included 304 patients with rheumatic diseases with a mean age of 44.9±12.9 years, disease duration 9.1±6.8 years, and age at onset 35.7±12.3 years. They were 85.5% females and 14.5% males with a family history of associated rheumatic disease in 28.3%, and positive consanguinity of 29.6%. 43.8% were active or passive smokers. Comorbidities included diabetes mellitus in 12.8%, hypertension in 11.8%, bronchial asthma in 1.6%, and hypothyroidism in 1%. Cardiovascular involvement was present in 11.5%, and none had any form of cancer.

Among all patients, 207 (68.1%) were diagnosed with RA, followed by 40 (13.2%) with SLE, and 23 (7.6%) were diagnosed with SPA. The rheumatoid factor (RF) was positive in 33.2%, the anti-cyclic citrullinated peptide (anti-CCP) in 10.2%, the antinuclear antibody in 17.4% and the anti-double stranded antibody deoxyribonucleic acid (anti-ds-DNA) in 8.6%. The mean RBCs was 4.6±1.4 x10^9^/mm^3^ (2.8–17.7 x10^9^/mm^3^), the hemoglobin (Hb) content was 12.02±1.7 g/dl (7.8–17.5 g/dl), the total leucocytic count (TLC) was 6.7±2.7 x10^3^/mm^3^ (2.7-21 x10^3^/mm^3^) and the platelets was 281.5±90.1 x10^3^/mm^3^ (67-599 x10^3^/mm^3^). The mean erythrocyte sedimentation rate (ESR) was 37.7±22.9 mm/1^st^hr (3–120 mm/1^st^hr).

The most frequent blood groups of the patients were as follows: group A in 37.8% (n=115), group B in 27.6% (n=84), group O in 19.1% (n=58), and group AB in 15.4% (n=47). Also, 271 (89.1%) patients were Rh positive and 33 (10.9%) were Rh negative.

### Association between rheumatic diseases and ABO and Rh groups

**Figure 1.**
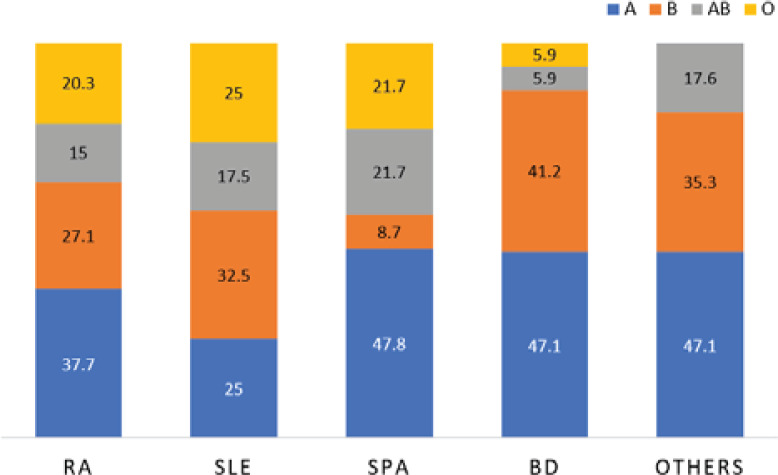
Frequency of distribution of ABO blood groups among different rheumatic diseases. ^$^Others includes: SSc: systemic sclerosis, IIM: idiopathic inflammatory myositis, SS: Sjögren’s syndrome, JIA: juvenile idiopathic arthritis, UCTD: undifferentiated connective tissue disease, and Vasculitis; polyarteritis nodosa and giant cell arteritis.

The characteristics of the studied rheumatic diseases patients according to the ABO and Rh blood group are presented in **Table 1**. While the B blood group was the most prevalent among patients with SLE (32.5%), the A and O blood groups were equally distributed (25%). The A blood group was significantly more prevalent in patients with RA (p=0.01), **Figure 1**. There was no significant difference between patients with various rheumatic disease and blood groups (p= 0.46).

**Table 1.** Distributions of the studied rheumatic diseases patients according to the ABO and Rhesus blood group.

Rheumatic Disease	N (%)	ABO blood systems in each disease					Rh
		A	B	AB	O	p-value	positive
RA	207 (68.1)	78 (37.7)	56 (27.1)	31 (15)	42 (20.3)	**0.014**	187 (90.3)
SLE	40 (13.2)	10 (25)	13 (32.5)	7 (17.5)	10 (25)	**0.004**	34 (85)
SPA	23 (7.6)	11 (47.8)	2 (8.7)	5 (21.7)	5 (21.7)	0.06	22 (95.7)
BD	17 (5.6)	8 (47.1)	7 (41.2)	1 (5.9)	1 (5.9)	0.11	15 (88.2)
Other^$^	17 (5.6)	8 (47.1)	6 (35.3)	3 (17.6)	0 (0)	-	13 (76.5)
Total N (%)	304	115 (37.8)	84 (27.6)	47(15.4)	58 (19.1)		271 (89.1)
p-value		0.46					0.21

RA: rheumatoid arthritis; SLE: systemic lupus erythematosus; SPA: spondyloarthritis; Behçet’s disease (BD).

$ Includes: SSc: systemic sclerosis, IIM: idiopathic inflammatory myositis, SS: Sjögren’s syndrome, JIA: juvenile idiopathic arthritis; UCTD: undifferentiated connective tissue disease, and Vasculitis; polyarteritis nodosa, and giant cell arteritis.

When rheumatic diseases were evaluated in terms of the distribution of the Rh blood group, Rh (+) was more prevalent in all rheumatic diseases, although there was no significant difference between the groups of rheumatic diseases in terms of Rh blood group distribution (p=0.21) (**Table 1**).

### Association between rheumatic diseases phenotypes and ABO and Rh groups

Comparison of the patients features according to the ABO system is presented in **Table 2**. Gender distribution (F:M) was significantly different between blood group A and O types (p=0.02). Positive smoking status was significantly different between all blood groups (p=0.03), with the main significance was between B vs O blood groups (p=0.006). In addition, positive anti-CCP and anti-dsDNA was different between different blood groups. The mean ESR level was more likely higher in blood groups B and O, but it did not reach statistical difference. According to the results of logistic regression analysis with type of rheumatic diseases, gender, smoking, and anti-CCP as independent variables, demonstrated that there was no significant relationship among different blood groups and any of the rheumatic diseases (p=0.074). However, female gender remained significant (p=0.016).

**Table 2.** Comparison of the patients’ characteristics according to the ABO system.

**Parameter mean ± SD n (%)**	**ABO blood groups in RD patients**				**p-values**						
	**A (115)**	**B (n=84)**	**AB (n=47)**	**O (n=58)**	**A vs**			**B vs**		**AB vs O**	**All groups**
					**B**	**AB**	**O**	**AB**	**O**		
Age (years)	45.8±12.9	442±12.8	45.4±13.6	43.9±13.2	0.33	0.87	0.37	0.56	0.97	0.57	0.71
F:M	4.5:1	5:1	8.4:1	13.5:1	0.66	0.15	**0.02**	0.33	0.07	0.51	0.14
DD (years)	9.5±6.9	8.9±6.9	7.9±6.1	9.7±7.1	0.55	0.13	0.91	0.36	0.55	0.17	0.49
Age Onset	36.1±11.9	35.1±10.8	37.4±14.2	34.3±13.5	0.54	0.58	0.28	0.34	0.71	0.26	0.59
Family hx	34 (29.6)	24 (28.6)	13 (27.7)	15 (25.9)	0.88	0.81	0.61	0.91	0.72	0.84	0.96
Consanguinity	37 (32.2)	22 (26.2)	11 (23.4)	20 (34.5)	0.36	0.25	0.76	0.73	0.3	0.21	0.5
Smoking	54 (47.0)	28 (33.3)	18 (38.3)	33 (56.9)	0.05	0.31	0.22	0.58	**0.006**	0.06	**0.03**
Rhesus	101 (87.8)	74 (88.1)	43 (91.5)	53 (91.4)	0.95	0.48	0.46	0.53	0.52	0.9	0.84
HTN	11 (9.6)	10 (11.9)	6 (12.8)	9 (15.5)	0.6	0.57	0.29	0.89	0.55	0.69	0.72
DM	19 (16.5)	10 (11.9)	4 (8.5)	6 (10.3)	0.35	0.14	0.25	0.53	0.77	0.75	0.47
CVD	12 (10.4)	9 (10.7)	8 (17.02)	6 (10.3)	0.95	0.3	0.9	0.34	0.94	0.33	0.65
Infection	5 (4.3)	5 (6)	4 (8.5)	3 (5.2)	0.62	0.36	0.81	0.6	0.84	0.51	0.77
RF	34 (29.6)	30 (35.7)	18 (38.3)	19 (32.8)	0.31	0.3	0.67	0.85	0.64	0.56	0.65
Anti-CCP	11 (9.6)	10 (11.9)	8 (17.02)	2 (3.4)	0.58	0.23	0.1	0.45	**0.049**	**0.03**	0.13
ANA	18 (15.7)	16 (19.04)	10 (21.3)	9 (15.5)	0.49	0.42	0.98	0.81	0.54	0.46	0.78
Anti-dsDNA	5 (4.3)	11 (13.1)	5 (10.6)	5 (8.6)	**0.04**	0.21	0.31	0.66	0.38	0.73	0.16
RBCs	4.4±0.64	4.7±0.82	5.02±2.6	4.3±0.53	0.06	0.23	0.8	0.55	0.06	0.21	0.22
Hb	12±1.75	12.1±1.92	12.2±1.51	11.7±1.3	0.77	0.58	0.28	0.81	0.21	0.13	0.59
TLC	6.7±2.8	6.7±2.81	6.8± 2.3	6.7±2.7	0.97	0.86	0.9	0.9	0.9	0.88	0.9
Platelets	272.7±82.1	296.3±108.3	286±83.6	270.6±78.3	0.19	0.46	0.9	0.63	0.21	0.45	0.46
ESR	31.3±21.1	39.2±24.7	30.7±19.9	39.4±26.1	0.08	0.89	0.17	0.1	0.9	0.17	0.16

RD: rheumatic disease; F:M: female:male, DD: disease duration; HTN: hypertension; DM: diabetes mellitus; CVD: cardiovascular disease; RF: rheumatoid factor; anti-CCP: anti-cyclic citrullinated peptide; ANA: anti-nuclear antibodies; anti-DNA: double stranded deoxyribonucleic acid; RBCs: red blood cell count (x10^9^/mm^3^); Hb: haemoglobin (g/dl); TLC: total leucocytic count (x10^3^/mm^3^); ESR: erythrocyte sedimentation rate (mm/1^st^hr).

## DISCUSSION

This study for the first time examined the relationship between rheumatic diseases and ABO and Rh blood groups in 304 patients in Egyptian population and determined whether blood group per se is associated with a specific disease and disease phenotype. The present study found that the blood group A and Rh (+) were more prevalent in patients with inflammatory rheumatic disease followed by B, O and AB blood groups, in respective order. Even though, the current study no significant difference in the distribution of ABO blood groups between patients with inflammatory rheumatic disease, the A and B blood groups were the most prevalent among patients with RA and SLE respectively. Moreover, we found that female gender, smoking and anti-CCP but not the blood groups are associated with type of rheumatic diseases. In the present study the distribution of blood groups was in harmony to the results of a recent Turkish study^2^ on rheumatic disease patients although the frequency of O type was higher than those in the current cases. The distribution of blood types in the current study; A>B>O>AB were comparable to the healthy Egyptian population distribution of A>O>B>AB and Rh+>Rh–.^17^ In this work the A blood type was more common in RA, JIA, SSc, SpA, and BD patients as in Çildağ et al. study.^2^ The most common blood type for FMF, SLE, SSc, and SS was type O, but AB in a similar Turkish work.^2^ The blood type A was highest in patients with erosive arthritis such as RA and AS, while blood type O was highest among patients with connective tissue disorders as SLE, SSc, and SS. The reason for this could be the difference in the genetic characteristics of the diseases.^2^ (2). Sika-Paotonu et al. reported that patients with blood group O have a lower risk for rheumatic mitral stenosis.^18^ Cohen et al. concluded that there was a significant correlation between the absence of the Rh antigen D and rheumatic disease.^19^

Genetic factors may play a role in the development and prognosis of certain diseases. Blood groups are inherited and are not affected by environmental factors.^20^ Blood groups have been evaluated as haematological markers in various studies, and clinical studies have demonstrated a relationship between the ABO and Rh blood groups and various types of cancer, diabetes, and cardiovascular diseases.^3^ Previous clinical studies have shown that various genetic and environmental factors play a role in the aetiology of inflammatory rheumatic disease.^2^ HLA genes, in particular, have been implicated in the pathogenesis of several rheumatic diseases (i.e. HLA-DRB1 and HLA-DP1 in RA, HLA-B27 in SPA, and HLA-B51 in BD. On the other hand, there are studies in literature identifying a possible relationship between HLA and ABO antigens.^21^

Although there are some reports indicating the absence of such relationship,^22^ we evaluated the association between the blood group antigens and demographic, clinical and laboratory manifestations. The interaction between blood group and sex has been previously observed in patients with persistent antiphospholipid antibodies.^23^ The present analyses add to these previous findings of the interaction between gender and rheumatic disease, indicating individuals with blood group O and a medical history of cancer had higher odds of developing thrombosis, thromboembolism or PE compared with blood group A or A + B combined. As plasma Von Will-ebrand factor (VWF) levels depend on the effect of ABO group, their levels were found to be 25%–30% lower in group O subjects than in non-O individuals.^24^ Moreover, in an Arab population, females had significantly lower levels of VWF which could indicate that females are more prone to bleeding. Only group O females had significantly lower VWF levels than non-O females.^25^ However, the mechanism underlying the interaction between blood group and gender in rheumatic diseases requires further research.

Our analyses indicated a high risk of smoking in individuals with all blood groups, and in particular, between blood group B compared to blood group O (p=0.006). Smoking is an established predictor for the development and severity of RA with prominent production of cytokines^26^ and is an independent risk factor for vascular thromboembolism (VTE).^27^ Interestingly, in the studied patients, smoking was more frequent among those with O blood type which could explain the absence of any reported thrombotic events among the various diseases. Persons with non-O blood type had a higher risk for development of VTE over persons with a blood type O. Heavy smoking was responsible for a further rise in the risk of VTE.^27^ It has been reported that the proportion of smokers is lower in A phenotype than in other blood types among older women.^28^ In an earlier study, there was a tendency to increased smoking in those with blood type B which is in hand with the results in the current work.^29^ On the other hand, others found no significant relationship between smoking habits, quantity of consumption and a particular ABO or Rh blood group.^30^ Further analysis on the smoking severity and history is recommended to confirm the relation.

Along with covering the surface of red blood cells, blood group antigens are found ubiquitously in the body, especially the parts which have an important role in the elimination process of infectious agents such as respiratory tract.^31^ Studies suggest that inflammatory response to certain infectious agents may vary according to the blood group antigens.^3,31^ Antibody detection and identification are processes that are commonly performed before transfusion of allogeneic RBCs.^32^ The ABO-specific autoantibodies thus appear similar in reactions and clinical manifestations to autoantibodies in general.^33^ Interestingly, AB blood group antibodies cross react with primate salivary ducts and may produce false positive anti-salivary duct autoantibodies staining in Sjögren’s syndrome and RA.^34^

The major strengths of this study were the large sample size, variety of examined diseases, and disease features. However, this study has some limitations. Our data is applicable only to the rheumatic diseases’ patients, with no comparison to healthy controls. Further studies on relation to disease activity and damage are encouraged. In conclusion, this work identified connections between the ABO blood group system with smoking in individuals with rheumatic diseases. Nevertheless, the present study provides an elegant overview for future research possibilities. In addition, it might be of interest to take sex differences into account because we observed interactions between sex and blood groups and autoimmune rheumatic disease.
